# P-228. A Multidisciplinary Approach to Reducing Central Line-Associated Bloodstream Infections (CLABSI)

**DOI:** 10.1093/ofid/ofae631.432

**Published:** 2025-01-29

**Authors:** Katherine A Rhodes, Bryan Youree

**Affiliations:** Texas Health Southwest, Fort Worth, Texas; Texas Health Southwest, Fort Worth, Texas

## Abstract

**Background:**

Our acute care hospital experienced 17 CLABSI events during 2021. This was a significant increase over 2020 and well above typical performance. The COVID-19 pandemic negatively impacted adherence to best practices. Point prevalence studies pointed to continued issues surrounding line maintenance. Gaps included inconsistent dressing care and site assessment, lack of compliance with scrubbing the hub prior to access, inconsistent use of chlorhexidine bathing, inadequate line securement, inadequate flushing of lines, inconsistent onboarding of new RNs, and missed daily review of necessity. The facility has initiated ongoing education & training, tracer observations, and recommended best practices. Each CLABSI event is estimated to cost $45,814 with 10.4 avoidable days and a 30% increase in mortality. It is considered a preventable healthcare-associated infection (HAI). A multidisciplinary approach was implemented in late 2021 with a stated goal of maximum 2 CLABSI events per year.

**Figure:**
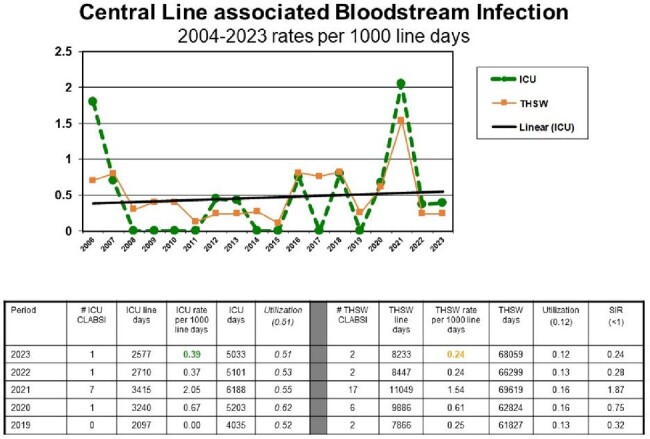

**Methods:**

CLABSI events are identified through daily review of blood culture reports by Infection Prevention. Events are defined using CDC NHSN criteria for CLABSI. Rates are calculated by number of events per 1000 device days and compared to national benchmarks and internal trending. A Standardized Infection Ratio (SIR) is utilized for external benchmarking.

Highlights of actions taken to drive improvement include:

- Creation of unit Central Line Champions to perform tracers and education
- Development of an Aseptic Access Tracer to standardize line maintenance
- Implementation of a biweekly Central Line Huddle led by VAT to facilitate timely line removal when no longer indicated
- Standardization of onboarding for new RNs to include line care and maintenance with a competency check off by VAT
- Feedback on strategies and data provided to key stakeholders

**Figure:**
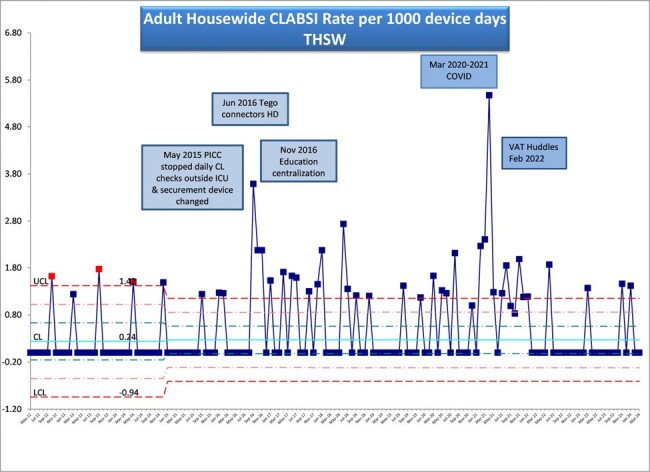

**Results:**

- CLABSI events were reduced from 17 events to 2 events in 2022 and sustained through 2023.
- CLABSI rates were reduced from 1.54 per 1000 line days to 0.24. SIR was reduced from 1.87 to 0.24.
- The Central Line Huddle facilitated early removal of 44 unnecessary central lines.
- Aseptic Access Tracer compliance was improved from 40% to 96% and sustained.

**Conclusion:**

A multidisciplinary approach was successful in improving CLABSI outcomes.

**Disclosures:**

**All Authors**: No reported disclosures

